# Relationship Between Sport Expertise and Postural Skills

**DOI:** 10.3389/fpsyg.2019.01428

**Published:** 2019-06-25

**Authors:** Thierry Paillard

**Affiliations:** Laboratoire Mouvement, Equilibre, Performance et Santé (UPRES EA 4445), Département STAPS, Université de Pau et des Pays de l’Adour, Tarbes, France

**Keywords:** balance, posture, postural control, sport, expertise, postural performance

## Abstract

The review addresses the relationship between sport expertise (i.e., sport competition level), postural performance (amount of motion of the center of mass/of pressure of foot or ability to preserve body balance), and postural strategy (geometric organization of different body segments as well as neurobiological involvement of organism). Since the conditions of postural evaluation are likely to influence results, the aim is to compare athletes at different competition levels in ecological postural conditions (specific postural conditions related to the sport practiced) and non-ecological postural conditions (decontextualized postural conditions in relation to the sport practiced). Evidence suggests that the most successful athletes in terms of sport competition level have the best postural performance both in ecological and non-ecological postural conditions. However, in non-ecological conditions, the postural tasks should be preferentially challenging or relatively close to the sport practice stance. Moreover, the most successful athletes also have more elaborate postural strategies compared with athletes at lower competition level. Mechanistic explanations as well as conceptual models are proposed to explain the role of different factors influencing the relationship between sport expertise and postural performance and strategy.

## Introduction

An expert athlete can be defined as a specialist in a particular sport who is able to achieve high levels of motor skills related to the sport practiced. His/her expertise requires the expression of physical qualities related to biomechanical (e.g., muscle strength, power, and segmental mobility), bioenergetic (energy supply), and/or bio-informational (information taking, reaction time, and response accuracy) aspects that are skillfully exploited in order to accomplish efficient motor command and control. In fact, his/her motor expression achieves maximal efficiency for minimal effort. The motor expression is based on phases of movement and balance whatever the sport practiced (i.e., any kind of sport technique carried out), however complex it may be. Indeed, a motor activity as basic as walking includes phases of monopedal and bipedal balance as well as phases of movement (swing) at the lower limb level (Bessou et al., 1988).

When movement is described in terms of efficiency or aestheticism in the sports community (by athletes, trainers, and the media), balance is rarely taken into account in the commentary/analysis of performance (Masseglia, 2011). However, movement and balance are intimately linked and inseparable when analyzing performance for most sport activities since no sport technique movement is (efficiently) achievable without an efficient body balance (Paillard, 2017a). Indeed, the slightest segmental movement engenders the displacement of the center of mass (COM), which means that movement potentially generates body imbalance which needs to be restored to avoid falling (Paillard, 2017b). Hence, during the sport practice, movement is continuous which compels the athlete to continually restore his/her balance through compensatory postural adjustments. In expert athletes, postural regulation is even anticipated before the onset of movement by anticipatory postural adjustments (Paillard, 2017a).

Before running, jumping, throwing, or any kind of motor action, each athlete must be able to maintain his/her balance (i.e., maintenance of the vertical projection of his/her center of mass above the base of support) and his/her posture (i.e., position of different body segments) in static condition (the base of support does not deform or move) or dynamic condition (the base of support is deformed and/or displaced) in order to not only resist falling but also to act efficiently (Paillard and Noe, 2015). An expert athlete showing high motor skills (e.g., in terms of accuracy, precision, agility, and velocity) in the expression of his/her motricity necessarily needs high postural skills (i.e., high ability to maintain balance in different postures when stationary or moving in the most economical way possible), particularly in the specific positions related to his/her sport (Asseman et al., 2008). Hence, evidence suggests that the skill level related to movement should be associated to the skill level related to balance in athletes. More precisely, there should be a relationship between sport expertise and postural skills for a given attainment level in a particular sport.

However, even if postural skills are fundamental to the use of motor skills, no proof of a direct relationship (i.e., the greater the sport competition level, the better postural skills; or better athletes also have better postural skills) has been established to date (Paillard, 2014, 2017a). It is not yet established that there is a close relationship between the motor expertise level (often analyzed through the sport competition level, e.g., amateur, local, regional, national, sub-elite, professional, international, and elite) and the postural skill level within the same sport, excluding comparison between different sports since each sport develops specific postural abilities (Paillard, 2014). Although a number of observational studies have analyzed this relationship, the resulting data are still subject to question. Hrysomallis (2011) carried out a significant review dealing with balance ability and athletic performance on the basis of quantitative analysis of postural skills (i.e., displacement of the center of foot of pressure – COP). However, the comparison of postural conditions related, or not related, to the sport practiced, i.e., ecological (specific postural condition) versus non-ecological (decontextualized postural condition) conditions, was not considered, although this seem likely to influence the relationship between the motor expertise level and the postural skills level. Indeed, in ecological condition, such as gymnasts executing handstands or rifle shooters accomplishing shooting while measuring the displacements of their COP, the possible relationship between motor (sport) expertise and postural skills could be strong since the amplitude of body sway (in stationary situations) is a criterion of performance in gymnastics (depending on the evaluation of judges) and the body segmental movements can be transmitted to the rifle barrel at the moment of shooting which are likely to affect performance (accuracy of shot) in rifle shooting. By comparison, in non-ecological or decontextualized postural conditions, such as for instance, bipedal quiet stance, for all kinds of athletes, this relationship between motor expertise and postural skills could be weaker or nonexistent, since there would not necessarily be any interaction between them. Moreover, qualitative data of postural skills could also deepen the knowledge relating to the relationship between the motor expertise level and the postural skills level by analyzing and describing how postural skills are organized in relation to the mechanical (geometric organization of different body segments) and neurophysiological (neurobiological involvement of organism) aspects.

The refinement of the method of analysis of the relationship between sport expertise and postural skills, based on the postural conditions in the sports studied supplemented by qualitative data of postural skills, could improve the analysis of sport performance and the elaboration of sport training. The present work therefore aims to provide an overview of the relationship between sports expertise and postural skills by identifying new mechanistic explanations.

## Methods for Studying the Relationship Between Motor Expertise and Postural Skills

**Figure 1:**
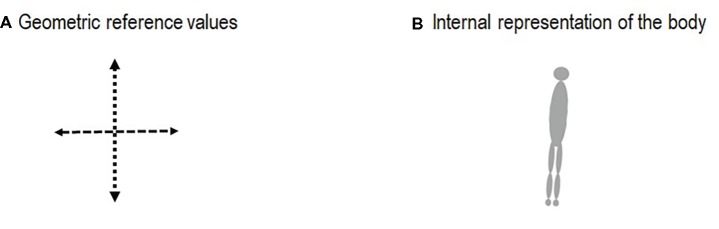
Posture and balance are regulated on the basis of reference values [**(A)**: geometric position and orientation of the segments that serve as a reference frame for perception and action with respect to the environment, i.e., knowledge of the orientation of the body axis and verticality] and internal representation of the body [**(B)**: also named body representation in space or postural body scheme].

Postural regulation is considered in the present manuscript as a motor action that involves taking information (sensory functions), processing this information, and command of action (central integration and command) as well as motor execution (motor function). It relies on neurophysiological components (the neural loops involved as well as their sensory receptors), cognitive function relative to body representation in space (cortical regulation), and motor function (muscle command). In this context, posture and balance are regulated on the basis of reference values (position and orientation of the segments that serve as a reference frame for perception and action with respect to the environment) and internal representation of the body (also named body representation in space or postural body scheme) (Figure 1). Actions require matching multisensory inputs regulating orientation and stabilization of body segments with anticipated and compensated postural adjustments (postural responses and motor programs) (Massion, 1994). In order to analyze the influence of these different components in the regulation of posture and balance, quantitative and qualitative measures are required.

### Quantitative and Qualitative Analyses

In each postural condition, postural skills can be quantified to assess postural performance not only by measuring the movement of the COM, the COP, and body segments but also by measuring electromyographic activities and evaluations of the contribution of different sensory information (i.e., visual, vestibular, proprioceptive, and cutaneous) in the participation of postural regulation. Postural skills can also be considered qualitatively to assess postural strategy by analyzing and describing how postural balance is organized in relation to the mechanical and neurophysiological aspects (Paillard and Noe, 2015). Postural performance combined with postural strategy is the main component of postural skills.

### Postural Performance and Strategy

In static condition, postural performance can be equated to the ability to minimize body sway (i.e., displacements of the COM – or displacements of the whole body – and/or displacements of the COP – or variation of the moment around the ankle) in conventional postural conditions (e.g., bipedal quiet stance). It can also refer to the ability to maintain body balance in challenging postural conditions with small bases of support (e.g., a stance classed as a handstand, monopedal stance) to avoid falling or/and postural imbalance (Paillard and Noe, 2015). In dynamic condition, postural performance can be equated to the ability to maintain body balance in changing postural conditions (e.g., displacement of pedal supports and displacement of the base of support), as well as in external mechanical changes (e.g., fast horizontal accelerations of the ground surface, unexpected percussion, or pushing a large body segment requiring postural reactions) in order to avoid falling (Paillard and Noe, 2015). Postural strategy can be defined on the basis of the spatial and temporal organization of different body segments as well as the extent and order of recruitment of different muscles activated. The different sensory inputs involved in postural regulation as well as the relative importance of different sensory information and/or the preferential involvement of different neuronal loops can also contribute to postural strategy.

### Methodological Caution

Movement can be characterized by whole body displacements or body segmental displacements in space. These displacements involve large and small accelerations and decelerations over large and small amplitudes. Body segments can alternate phases of long displacements with phases more or less stationary. During phases of long displacements, postural skills are used particularly in dynamic condition while during stationary phases postural skills are mainly used in static conditions but both phases follow each other continually. Hence, the separate analysis of movement and balance is mechanically very complex. As mentioned above, movement and balance are intimately linked and inseparable when analyzing motor expertise (e.g., performance) for many sports.

### Evaluation Conditions

For a given sport, motor expertise could greatly depend on postural skills while postural skills could also depend on motor expertise. Both skills can be reciprocally influenced but it is difficult to determine precisely the influence of each one, particularly in a very mobile sport (constant and long displacements). As part of mobile sports, the analysis of the relationship between motor expertise (e.g., performance) and postural skills cannot be directly quantified since balance and dynamic movement are almost inextricable. Hence, one can only attempt to correlate/associate postural skills with motor skills through separate tests evaluating each of the two skills. In this case, the evaluation of postural skills can only be undertaken in non-ecological conditions, i.e., in a decontextualized environment in relation to the sport practice. However, for a static or low mobility sport activity (no significant displacements), the postural skills and motor expertise can be simultaneously quantified. In this case, the evaluation of postural skills can be made in ecological conditions, i.e., during the sport practice (contextualized environment).

Knowing that each sport develops specific postural skills (Paillard, 2014), the study of the relationship between sport expertise and postural skills should only include young adult athletes (in order to avoid the effects related to age, i.e., development in children and involution in aged subjects) who practice the same sport. Sport experts are elite athletes compared to sub-elite, amateur, or recreational athletes, who are not considered as sport experts but as non-elite athletes.

## Postural Performance and Sport Performance

The quantitative analysis of postural skills helps to define the postural performance of athletes while their sport performance is established through their sport competition level. Since postural performance can be studied in ecological and non-ecological postural conditions (specific condition versus non-specific or decontextualized condition) in relation to the considered sport, it is worth studying the relationship between postural performance and sport performance in these two postural conditions.

### Ecological Postural Conditions

In ecological conditions, athlete’s postural skills are evaluated during their practice of the sport (e.g., rifle shooting, recurve archery) or during the execution of specific motor skills (e.g., handstand posture in gymnasts, juggling in jugglers). With sports such as rifle shooting and recurve archery, one could naturally think that body sway at the moment of shooting would affect its accuracy. Hence, it seems relevant to evaluate the relationship between sport performance and postural performance during shooting. It was observed that the best elite rifle shooters and archers also had the best postural performance (Ball et al., 2003; Musa et al., 2018). By holding their rifles with their arms, rifle shooters create a trunk-head-arms block that must be well interlocked to limit the movements of the rifle and to enable the best possible shooting score (Bermejo et al., 2015). Hence, Ball et al.’s result is not surprising since body sway (evaluated through displacement of COP) is likely to be transmitted to the motion of the gun and aim point and is thus likely to affect shooting performance. The average magnitude of displacement of COP would be smaller before high shooting scores than before low shooting scores (Konttinen et al., 1999). Obviously, shooting accuracy depends on body sway while the ability to keep the gun stable is a sine qua none condition for high shooting performance (Mononen et al., 2007). Indeed, the reduction of body sway alone is not a guarantee for improving shooting performance if it is not (necessarily) associated to minimal movement of the gun barrel (Konttinen et al., 1999). This explains why postural performance would have a limited direct influence on shooting score (in terms of variance of shooting score) but would have a real but indirect influence on shooting score through the ability to hold more stably (Ihalainen et al., 2016, 2018). This principle was corroborated by Ko et al. (2018) who showed that the amount of the COP and pistol motion was lower for skilled pistol shooters than novice shooters. Postural regulation was better coordinated with arm movements to minimize the motion of the pistol in skilled shooters than in novice shooters (Ko et al., 2017).

Moreover, the postural performance level would reflect the sport performance level of rifle shooters since international level rifle shooters were more stable than national level shooters who were more stable than amateur shooters (Era et al., 1996). These authors specified that elite shooters were able to reduce their body sway in the last few seconds just before shooting while amateur shooters kept their body sway pattern during the same time. Moreover, expert shooters limit/control movement in the medio-lateral direction more than shooters without experience (Niinimaa and McAvoy, 1983). In fact, international shooters were able to reduce medio-lateral movements more throughout the test period and antero-posterior movements more during the last few seconds before shooting than national shooters (Ihalainen et al., 2016). COP measurements carried out 1 s prior to arrow release and 0.5 s post-arrow release with elite archery shooters confirmed that reduced postural sway was a predictor of higher scoring shots (Spratford and Campbell, 2017).

Ecological postural tasks performed in dynamic condition (i.e., in movement) such as ball juggling tasks are also of use to study the relationship between motor performance and postural performance using simultaneous measures. Expert jugglers showed better postural performance than intermediate jugglers which corroborated the idea that juggling skills are associated with body sway (Rodrigues et al., 2016). In addition, these authors observed that expert jugglers were less affected by the reduction of the basis of support during juggling tasks than intermediate jugglers. Moreover, the execution of gymnastic tasks by expert and non-expert gymnasts while measuring body sway with force platform can increase our understanding of this subject. The handstand and monopedal stance (but not the bipedal quiet stance) differentiates postural performance between expert gymnasts and non-expert gymnasts (Asseman et al., 2005, 2008; Marcolin et al., 2019). These authors reported that the more the postural tasks are specific and difficult, the more the athletes’ postural performance can be related to their competition level. Other works corroborate this principle since dance-like postural tasks have made it possible to discriminate expert dancers from intermediate dancers while this was not possible though static postural tasks (Munzert et al., 2018). Paillard et al. (2011) observed that surfers at national and international competition levels were distinguished from surfers at local competition level when using dynamic postural tasks carried out on an unstable support (very close to the postural condition implied by surfing) but not when using static postural tasks completed on a stable support.

Evidence suggests that in ecological postural condition there is a relationship between postural performance and sport performance.

### Non-ecological Postural Conditions

For non-ecological postural tasks such as bipedal quiet stance or any other postural stance decontextualized in relation to the sport considered, the relationship considered was largely studied through a number of sport activities whether mobile or immobile. In an immobile sport activity, without holding a rifle and without specific clothing (i.e., non-ecological condition), elite shooters (competition shooters) had better postural performance than non-elite (military) shooters in visual and nonvisual conditions (Aalto et al., 1990). In mobile sports, there would be also a relationship between sport performance and postural performance in athletes practicing different activities such as soccer, gymnastics, golf, baseball, and stand-up paddle board (Paillard et al., 2006, 2007b, 2011; Paillard and Noe, 2006; Sell et al., 2007; Asseman et al., 2008; Butler et al., 2016; Schram et al., 2016; Jadczak et al., 2018; Pau et al., 2018). However, for a given sport, it is not always possible to distinguish the athletes’ postural performance according to their competition levels in the standard postural conditions (e.g., bipedal quiet stance) often used by experimenters (Paillard et al., 2002; Asseman et al., 2004). Similarly, in static and non-specific conditions (i.e., non-ecological conditions), expert judokas and surfers did not exhibit better postural performance than non-expert judokas and surfers (Paillard et al., 2002; Chapman et al., 2008). In fact, postural tasks too simple and easy make it difficult to discriminate between the competition levels of athletes in terms of postural performance.

Nevertheless, in response to unexpected external disturbances, elite female ice hockey players showed shorter recovery period of COM and smaller body sway than non-athletes (Kim et al., 2018). Golfers and runners demonstrated faster onset of trunk muscle activation and higher muscle activation amplitudes in response to sudden trunk loading disturbances when compared to control subjects (Glofcheskie and Brown, 2017). Young soccer players at national level displayed faster and more efficient postural stabilization after a forward jump than young soccer players at regional level (Pau et al., 2018). On the basis of specific and difficult postural tasks, one can wonder whether the relationship between sport performance and postural performance is found in postural disturbance conditions particularly when activating, naturally or artificially, the sensory functions of athletes. As part of vestibular stimulation induced by several and successive body rotations related to dance figures, the COP displacements of elite dancers were smaller than those of amateur dancers and control subjects (Hopper et al., 2014). As part of artificial sensory manipulations at the level of plantar cutaneous (cooling the feet) and myotatic proprioceptive (electrically stimulating the lower limb musculature and blockage of cervical segment) information, professional soccer players who initially exhibited better postural performance (non-manipulated postural condition) than amateur soccer players also showed smaller COP displacements in the manipulated condition (Paillard et al., 2007b). Evidence suggests that whatever the postural condition – i.e., with or without disturbance – the most successful athletes (competition level) also display better postural performance.

Moreover, the conditions of practice of some sports are likely to impact negatively postural performance when they are intensively practiced (i.e., frequent practices). Indeed, at the end of a sports season, skiers competing at a national level showed worse postural performance than skiers competing at a regional level (Noé and Paillard, 2005). On the basis of data mentioned above, this result is contradictory since Noé and Paillard (2005) postulated that this phenomenon occurred because of the effects of wearing ski boots during frequent training sessions. Ski boots of Alpine skiing are rigid and limit/reduce ankle movement, so in the long run the frequent wearing of such ski boots would adversely affect proprioception and postural performance. Since national skiers spent more time at training than regional skiers, this would explain why their postural performance was worse than regional skiers at the end of sport season (Noé and Paillard, 2005).

In non-ecological postural conditions, the relationship between sport performance and postural performance is not systematically observed in athletes and requires caution in the methods of analysis.

### Summary

According to the current literature, the ecological postural condition turns out to be more appropriate to investigate the relationship between sport performance and postural performance. In non-ecological postural conditions, this relationship would assuredly be preserved only in two conditions. First, the postural tasks used for the evaluation should be sufficiently specific and difficult in relation to the sport considered, and second, material conditions of sport practice should not affect proprioception.

## Postural Strategy and Sport Performance

By describing postural strategy, i.e., how postural regulation is organized in relation to the mechanical and neurophysiological aspects (cf. the sub-section “Postural Performance and Strategy”), it turns out to be possible to compare expert or elite athletes with non-expert or non-elite athletes and to specify the differences between them from a postural view point.

### Ecological Postural Conditions

Postural strategy between expert athletes and non-expert athletes can be differentiated through biomechanical and neurophysiological analyses which focus on input of information (sensory functions), processing this information, and commanding action (central integration) as well as motor action (motor function).

Shooting and juggling tasks are well suited to our study framework since it is relatively easy to simultaneously evaluated motor performance and postural performance. Elite shooters gave less importance to visual cues and more importance to proprioceptive and vestibular cues than non-elite shooters (Aalto et al., 1990). In shooting conditions, the vision is devoted only to targeting and does not contribute to control of posture (Aalto et al., 1990). In this case, the reduction of the contribution of visual information in postural regulation is compensated for by greater contributions of proprioceptive and vestibular information (Aalto et al., 1990) which enables elite shooters to dedicate more resources to the main motor task (i.e., shooting) especially for controlling the rifle movement and pressing its trigger (Konttinen et al., 1999). Moreover, expert jugglers were less dependent on foveal vision than intermediate jugglers who moved their gaze around a larger visual area. This suggests that intermediate jugglers were searching spatially for the balls which would be associated with an increase of body sway (Rodrigues et al., 2016).

Konttinen et al. (1999) found that non-elite rifle shooters more actively controlled their posture (cerebral and muscle activities) during shooting than elite rifle shooters. Intermediate jugglers displayed a higher level of attention and prioritized the manipulation task rather than postural regulation, increasing body sway differently from expert jugglers, who were able to deal better with both the manipulation task and postural regulation (Rodrigues et al., 2016). A lower cognitive load for expert jugglers would use their resources less which could explain why they were less affected than intermediate jugglers since their adaptation capacities remained greater (Rodrigues et al., 2016). Moreover, high level biathletes would present better central integration of sensory cues and/or better filtering of erroneous cues than lower level biathletes (Simoneau et al., 1996). According to these authors, higher level biathletes would also detect the discrepancy between the actual posture and the desired posture more quickly while they would demonstrate better motor coordination and motor responses earlier than lower level biathletes.

In ecological postural conditions, on the basis of the data above, elite and non-elite shooters, biathletes, and jugglers would implement different postural strategies to regulate their posture.

### Non-ecological Postural Conditions

Biomechanical and neurophysiological analyses also highlighted differences in terms of postural strategies between elite and non-elite athletes in non-ecological postural conditions.

With decontextualized postural tasks in relation to the sports considered (e.g., bipedal stance), biomechanical data obtained with accelerometric measures showed that the number of mobilized body segments decreased as the sport level increased in judokas (Mesure and Crémieux, 1996). Moreover, with greater gymnastic skills, acceleration time series were less variable and more stable (Lamoth et al., 2009). This suggests that the best athletes in terms of sport level could proceed by slight (local) postural adjustments while the other athletes would undertake more global postural strategies as part of pure postural tasks. However, as part of more complex locomotor tasks (e.g., narrow and wide beam-walking) rather than pure postural tasks, Sawers et al. (2015) observed that expert ballet dancers used multiple motor modules for executing a biomechanical function whereas novice dancers executed such functions with a single motor module. Taken together, these results emphasize that the postural adjustment strategy between experts and non-experts could depend on the context of the motor task. Experts would have more flexibility in achieving motor goals thanks to a larger motor repertoire which would be useful under challenging conditions (Sawers et al., 2015). In turn, as part of easy or reflex postural conditions experts could operate through slight postural adjustments. Moreover, postural strategies could be more automatic in expert athletes (Lamoth et al., 2009; Sawers et al., 2015; Michalska et al., 2018) while they would be more actively controlled in non-expert athletes.

Neurophysiological data indicate that gymnast and soccer player experts made better use of vestibular inputs, probably through a better central integration, than gymnast and soccer player non-experts (Bringoux et al., 2000; Paillard et al., 2006). In expert athletes, a greater contribution of vestibular information would reduce the contribution of proprioception especially as part of easy and simple postural tasks. In this case, the proprioceptive function would be saved and would offer supplementary resources for carrying out more challenging postural tasks. Hence, expert athletes would have additional abilities to cope with destabilizing motor tasks (Paillard et al., 2006). By comparison, in non-expert athletes, as part of easy and simple postural tasks, the lower contribution of vestibular information would result in a greater contribution of proprioception which would already be fully exploited. Thus, the contribution of proprioception in postural regulation could not increase as the difficulty of the postural task increased and would not make it possible to cope with particularly challenging and destabilizing postural tasks (Paillard et al., 2006). Evidence suggests that the contribution of vestibular and proprioceptive inputs increases in postural regulation as the competition level is raised. In this context, it would be logical that the contribution of visual cues diminishes.

Indeed, the contribution of visual information is reduced as the sport competition level increased in surfers and soccer players (Paillard et al., 2006, 2011; Paillard and Noé, 2006; Chapman et al., 2008). The lower visual dependence in elite soccer players in comparison with sub-elite soccer players enables elite soccer players to devote their gaze more to processing the information that stems from the game (Paillard and Noé, 2006; Paillard et al., 2006; Zouita Ben Moussa et al., 2012). Sub-elite soccer players being more visually-dependent than elite soccer players would have less resources to devote to the game actions. Indeed, the analysis of movements of the ball and the team partners and opponents is essential in soccer in order to implement cooperative or opposition strategies. For a given soccer player, the time required to take in visual information needed to perform his/her own motor actions (i.e., balance, movement, displacement, control of the ball) reduces the time available for analyzing the game (Paillard and Noé, 2006). For instance, the control of the ball with the feet involves looking down which limits the observation time of the environment.

Moreover, it turns out that the less the visual contribution, the more the proprioception contribution in postural regulation by athletes. This phenomenon is particularly observed as part of the sport expertise (e.g., Paillard and Noé, 2006). Expert surfers shifted sensorimotor dominance from vision to proprioception (Chapman et al., 2008). Postural regulation achieved on the basis of low visual dependence would facilitate the technical expression and motor skills. This would explain why the visual dependence is lower in expert surfers than in non-expert surfers. Expert surfers would divert their visual contribution from postural regulation mainly to processing the relevant information about environmental factors such as height and shape of waves. As observed previously by Asseman et al. (2005) with elite and sub-elite gymnasts, Paillard et al. (2011) indicated that the difference in visual dependence between elite surfers and sub-elite surfers increased as the difficulty of the postural task increased (e.g., bipedal vs. monopedal, stable support vs. unstable support; upright vs. handstand postures).

It is however important to note that certain physical activities such as dancing develop postural and motor strategies which are more visually-dependent (related to the fact that the environment is stable) than most other activities such team sports or sliding sports as mentioned above (Michalska et al., 2018). This is why the contribution of visual information does not decrease as the expertise level is increased in dancers. The suppression of visual cues would penalize expert dancers more than non-expert dancers (Paillard, 2017a). The physical activities practiced in a stable environment and an unmoving space, such as dance sports, remain exceptions since generally in an unstable environment and/or space, the contribution of visual information decreases as the expert level is increased. Overall, sport skill-specific expertise impacts sensory integration for spatial referencing and postural skills (Thalassinos et al., 2018).

Moreover, the expertise level would also influence the motor command of the postural function. Kim et al. (2018) observed that non-athletes displayed more co-activation of the ankle plantar flexors and dorsiflexors while elite female ice hockey players showed low co-activation strategy of agonist and antagonist in ankle and neck extensors. Sawers et al. (2015) also found less muscle co-activation and greater efficiency in muscle output in expert ballet dancers than in novice dancers (no dance or gymnastic training).

In non-ecological postural conditions, the different sensory and motor resources are exploited differently in expert and non-expert athletes for regulating posture.

### Summary

Overall, for both ecological and non-ecological postural tasks, expert and non-expert athletes deploy different postural strategies whether for basic or challenging postural tasks.

## Postural Skills and Individual Natural Predispositions

It is known that motor performance depends on training (quantitative and qualitative aspects) as well as individual natural predispositions (psychological and physiological qualities adapted to the specific aspects of a given sport). While it is also known that training influences postural skills of athletes (Paillard, 2017a), little is known about the influence of individual natural predispositions on postural skills and this deserves to be studied.

If the individual natural predispositions can affect postural skills, they could act on the sensory, central and/or motor components of the postural function. At the sensory level, the proprioception emanating from lower and upper limbs would be more accurate in elite athletes than in sub-elite athletes independent of the amount of training (i.e., the number of years of practice) for different sports such as badminton, soccer, swimming (Han et al., 2015a). According to these authors, this result illustrates that the amount of training would not impact proprioception but individual predispositions would determine its accuracy. Even if caution is due, one can postulate that the amount of training would not be fundamental to the proprioception output.

**Figure 2:**
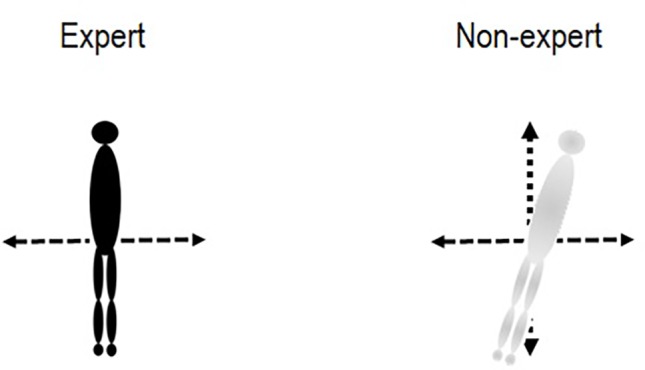
Comparison of abilities to regulate posture and balance between sportive non-experts and sportive experts. Experts would have a better knowledge of the orientation of the body axis and verticality as well as a more accuracy internal body representation than non-experts.

At the level of the central component of the postural function, Paillard et al. (2006) inferred that elite soccer players had a better knowledge of the orientation of body axis and verticality than sub-elite soccer players (Figure 2). This could mean more accurate internal body representation and subjective verticality in elite soccer players than in sub-elite soccer players. In addition, the reaction time (total reaction time and premotor reaction time) to sport-specific visual stimuli was shorter in elite taekwondo practitioners than in sub-elite taekwondo practitioners (Chung and Ng, 2012). Hence, these authors highlighted that the speed of perception would be faster to discriminate relevant cues in motor actions of opponents (especially sport-specific postures) in the elites taekwondo practitioners than in sub-elite practitioners.

At the level of motor output of the postural function, Barbado et al. (2016) reported higher trunk extensor muscle strength in elite judokas than in sub-elite judokas. On the basis of postural data, Paillard and Noé (2006) concluded that the tone of the posterior leg muscles would be greater in elite soccer players than in sub-elite soccer players. Moreover, the neuromuscular excitability threshold of the quadriceps muscles was lower in the elite taekwondo practitioners than in sub-elite taekwondo practitioners (Chung and Ng, 2012). As it is accepted that the higher the muscle excitability, the better the muscle contractility, these authors stated that the motor output of the postural function would be naturally better in elite taekwondo practitioners than in sub-elite taekwondo practitioners.

On the basis of the data above, it can be assumed that individual predispositions influence postural skills. Hence, one can wonder whether the individual postural potential can be improved thanks to sport training.

## Individual Postural Trainability

The athlete’s potential to increase his/her postural skills following training can be defined by the term “postural trainability”. The postural skills can initially (naturally) be low or high in athletes but with increased experience whatever their initial level (low or high), some athletes continue to progress while others stagnate. Hence, the postural trainability differs between athletes. On the basis of these practical observations that emanate from coaches (unpublished data) regardless of the initial natural predispositions, the individual trainability is more or less great and it is difficult to identify the subjects who can make great progress and those who cannot. According to current knowledge, whether for trainers (coaches) or scientists, individual trainability is difficult to estimate prospectively (predict) for a sports career. Future research works should explore individual postural trainability on the basis of interventional studies since the current literature is relatively devoid of useful data likely to advance the training and selection plans of young athletes.

## Transfer Between Postural Skills and Motor Skills

Another problem that presents itself to trainers is that the influence of postural skills on sport performance is currently still unknown. One can observe that the most successful athletes (in relation to the competition level) have the best postural skills but one does not know if those with the best postural skills are always the most successful athletes. Although there are relationships between postural skills and technical skills (dribbles, passes, shots) as well as running speeds (including accelerating, stopping) in small-sided soccer games (Edis et al., 2016, 2017), currently, the transfer of postural skills toward motor skills remains to be explored through sport training. As far as is known, there is no evidence that improvement in postural skills would enhance motor skills.

In turn, particular training leads to specific postural regulation induced by the acquisition of specific new motor skills due to the practice of the specific movements (Paillard et al., 2007a). This would support the idea that each learned new technique is associated with new postural adaptation/skills. However, if a transfer of motor skills toward postural skills occurs, it would be limited to very close movements in terms of posture/position compared to those practiced in training and competition (Paillard et al., 2017a). If it is not a skills transfer, the acquisition of new postural skills would be integrated into new motor skills which would mean that motor skills and postural skills are indissociable.

The state of the current literature suggests that studies should be undertaken in order to unravel the possible reciprocal influences between postural skills and motor (sport) skills. These works could also show that there is not any dependence between these two skills which would be surprising from a theoretical point of view, on the basis of current knowledges.

## Conclusion

Evidence suggests that the most successful athletes in term of sport competition level have the best postural performance both in ecological and non-ecological postural conditions. However, in non-ecological condition, postural tasks should be preferentially challenging or relatively close to the sport practice stance. The relationship between sport performance and postural performance is not systematic when the postural conditions are remote from those of the sport practice. Moreover, the most successful athletes also have more elaborate postural strategies compared with athletes at lower competition levels. Sports experts would also show better natural postural predispositions than sports non-experts. Currently, it is known that motor skills could greatly depend on postural skills while postural skills could also depend on motor skills. Both skills influence each other but it is difficult to determine precisely the influence of one over the other either for ecological and non-ecological postural tasks, even if the current assumption is that the influence of motor skills toward postural skills would probably be stronger than the reverse influence. Future studies should unravel the concepts involved thus enabling sports trainers (coaches) to improve their intervention and to refine their training programs for athletes as well as enabling physicians to enhance their preventive and therapeutic strategies.

## Author Contributions

The author confirms being the sole contributor of this work and has approved it for publication.

### Conflict of Interest Statement

The author declares that the research was conducted in the absence of any commercial or financial relationships that could be construed as a potential conflict of interest.
